# Virtual Exchange in Global Health: an innovative educational approach to foster socially responsible overseas collaboration

**DOI:** 10.1186/s41239-021-00266-x

**Published:** 2021-06-10

**Authors:** Keith Bowen, Michele Barry, Ashley Jowell, Diana Maddah, Nael H. Alami

**Affiliations:** 1 Learning Sciences & Technology Design Program at Stanford University, Stanford, USA; 2 Medicine and Tropical Diseases, Center for Innovation in Global Health, Global Health at Stanford University, Stanford, USA; 3 Stanford University School of Medicine, Stanford, USA; 4 School of Health Sciences at the Modern University for Business & Science, Beirut, Lebanon; 5 Modern University for Business & Science, Beirut, Lebanon

**Keywords:** Global Health, Refugees, Ethics, Sustainability, Virtual Exchange, Collaborative Learning, Virtual Reality

## Abstract

Educators who design and manage study abroad programs face a series of ethical responsibilities. Meeting these responsibilities is critical in the field of global health, where study abroad programs are often designed to provide healthcare services in under-resourced communities. Leaders in global health have thus formed working groups to study the ethical implications of overseas programming and have led the way in establishing socially responsible best practices for study abroad. Their recommendations include development of bidirectional programming that is designed for mutual and equitable benefits, focused on locally identified needs and priorities, attentive to local community costs, and structured to build local capacity to ensure sustainability. Implementation remains a key challenge, however. Sustainable, bidirectional programming is difficult and costly. In the present study, authors questioned how technology could be used to connect students of global health in distant countries to make socially responsible global health programming more accessible. Drawing on empirical research in the learning sciences and leveraging best practices in technology design, the authors developed a Virtual Exchange in Global Health to connect university students in the U.S. with counterparts in Lebanon, who worked in teams to address humanitarian problems in Syrian refugee camps. Early results demonstrate the value of this approach. At dramatically lower cost than traditional study abroad—and with essentially no carbon footprint—students recognized complementary strengths in each other through bidirectional programming, learned about local needs and priorities through Virtual Reality, and built sustaining relationships while addressing a difficult real-world problem. The authors learned that technology could effectively facilitate socially responsible global health programming and do so at low cost. The program has important implications for teaching and learning during the COVID-19 crisis and beyond.

## Introduction

COVID-19 has deeply disrupted teaching and learning in universities across the globe. In this period of forced experimentation, faculty and support staff work assiduously to recreate familiar classroom activities in virtual environments, sharing slide shows through web-conference tools, assigning small-group problems in virtual break-out rooms, and encouraging students to work collaboratively using shared online documents and virtual whiteboards. But technology can also be used to create innovative learning experiences that transcend what is possible in traditional brick-and-mortar classrooms. In this research, conducted before the pandemic, early adopters of technology used a range of methods to connect two courses in global health, one in the U.S. and the other in Lebanon, to foster socially responsible overseas collaboration, carefully guiding students as they addressed humanitarian problems in Syrian refugee camps. This global health programming has important implications for teaching during the pandemic and beyond.

### Short-Term Experiences in Global Health (STEGHs)

Rapid growth of interest in global health education has led to steady expansion of academic programs in the field (Drain et al., 2017). Because students seek direct experience in confronting and addressing global health challenges, program directors like to offer Short-Term Experiences in Global Health, or STEGHs, that involve crossing international borders (Crump et al., 2010; Melby et al., 2016). Consistent with the general literature in study abroad programs, researchers in global health have documented a number of beneficial outcomes of STEGHs, including measurable increases in the cross-cultural competence of students as well as greater interest in volunteerism and humanitarianism, which can affect students’ career choices (Godkin & Savageau, 2003; Gupta et al., 1999).

### Ethical responsibilities

Those who develop and manage programs to send students to low-income countries confront a series of ethical responsibilities associated with overseeing their work. Meeting these responsibilities is important in any field but is especially critical in the field of global health, where programs are often conceptualized and designed to provide various forms of healthcare service in under-resourced communities (Crump et al., 2010; Melby et al., 2016). For this reason, international leaders in global health have formed working groups to study the ethical ramifications of STEGHs (Crump et al., 2010; Melby et al., 2016). Their efforts have led the way in developing and establishing ethical best practices for study abroad. Key recommendations include development of bidirectional programming that is designed for mutual and equitable benefits, focused on locally identified needs and priorities, attentive to comprehensive costs to local communities, and structured to build local capacity to ensure long-term sustainability (Crump et al., 2010; Melby et al., 2016).

### Challenges in implementation

Implementation of programming to meet these requirements was a challenge before the pandemic and will remain so after, especially developing and funding bidirectional programming for students from both high- and low-income countries. In-person study abroad is expensive and demanding. It is much more accessible to students from high-income countries, and it requires even these students to have sufficient means and opportunity to live far from home for extended periods of time. Despite attempts by various institutions to increase participation in study abroad, including the U.S. Congress (McMurtrie & Bollag, 2007), programs have proven difficult to scale. In normal years, estimates of the proportion of students who study abroad range between 1 and 2%, and students with less economic, cultural, and social capital are least likely to participate (Centre for Educational Research & Innovation, 2010; Lipinski, 2014; Simon & Ainsworth, 2012). Programming that is unidirectional—focused exclusively on the needs of sending institutions in high-income countries—leads to imbalances and ethical problems, including development of interventions that do not fit local needs and priorities, are not sustainable, and do not account for full costs to local communities (Godkin & Savageau, 2003; Gupta et al., 1999). How can program designers address this difficult problem during the pandemic and beyond? This research postulates that through well-designed technological solutions, institutions of higher education can meet fundamental ethical requirements of global health programming at costs designed to scale.

## Methods

Drawing on empirical research in the learning sciences, including study of Contact Theory (Allport, 1954; Pettigrew & Tropp, 2006), Collaborative Learning (Roschelle & Teasley, 1995), and Problem-Based Learning (Barron et al., 1998; De Graaff & Kolmos, 2007), and leveraging best practices in technology design (Brown, 2008; Dorst, 2011), the Virtual Exchange program at Stanford University was conceptualized, designed, and launched to use new media and technology to reduce the cost of student exchange and in so doing to make its profound benefits more accessible (Bowen et al., 2019). In the present study, researchers asked whether, despite reduced cost and resource expenditures in implementation, Virtual Exchange could provide socially responsible, bidirectional programming in the field of global health, connecting global health students in the U.S. with global health students in Lebanon. Could technology facilitate learning in ways that go beyond what is possible in brick-and-mortar classrooms? Could technology offer scalable international programming that meets key ethical responsibilities of global health exchange? The sub-sections that follow describe the participants identified and selected for the study, along with the design of the program, data gathering procedures, and data analysis.

### Participants

The resulting Virtual Exchange in Global Health was conducted for 6 weeks in the fall of 2018. It was designed to connect 24 students enrolled in a global health course at Stanford University in the U.S. with 24 students of similar age enrolled in a comparable global health course at the Modern University of Business & Science in Lebanon, which also hosted students from the Lebanese American University and the American University in Beirut. In the U.S., 16 students were female, and 8 students were male. In Lebanon, 20 students were female, and 4 were male. Student ages ranged from 18 to 38. Most students on both campuses were in their early twenties and were undergraduates. In the U.S., most of the undergraduates studied Human Biology. In Lebanon, undergraduates were for the most part split between Biochemistry and Public Health. A few students in both countries were in graduate school or medical school. Graduate students on all campuses tended to have some practical experience in humanitarian aid.

### Program design

In-person student exchange programs were deliberately accelerated after World War II and during the Cold War to improve relations between historical and geopolitical adversaries (Atkinson, 2010; Bu, 1999). Since then, programs have been designed around Contact Theory (Allport, 1954; Pettigrew & Tropp, 2006), a conceptual foundation for intercultural exchange that for several decades has been carefully investigated in empirical studies and meta-analyses (Paige et al., 2009; Pettigrew & Tropp, 2006) and continuously refined in application by program developers. According to Contact Theory, when groups are largely unfamiliar with one another, come from substantially different socio-economic and cultural backgrounds, have potential suspicion of each other due to negative stereotypes—and may even be in open political, economic, or social conflict—contact between them can lead to beneficial outcomes, provided that the contact is structured in specific ways:

- Official approval: The contact should be officially approved on both sides.
- Social equity: The contact should be structured to promote equity in social status.
- Purposeful pursuits: The groups should engage in purposeful pursuits.
- Cooperation: Their efforts should be cooperative rather than competitive (Allport, 1954).

In this Virtual Exchange in Global Health, researchers and faculty leveraged Contact Theory and associated practices, including Problem-Based Learning, which focuses student efforts around a specific and complex problem (Barron et al., 1998; De Graaff & Kolmos, 2007), and Collaborative Learning, with its emphasis on student interaction in a Joint Problem Space (Roschelle & Teasley, 1995), to develop an innovative educational program powered by new technologies. The program began with introductions, shared lectures, and discussions over videoconference, which provided official approval and promoted social equity, and were designed to prepare students for a purposeful and collaborative small-group capstone assignment over web-conference. In the capstone, teams of six students, three from each country, worked together to identify, study, and address particular problems for refugees at a site in the Beqaa Valley in Lebanon, where the Modern University of Business & Science has been providing various forms of humanitarian assistance in partnership with UNHCR, aid agencies, national ministries, and local NGOs. Students analyzed conditions at the site through 360-degree videos with Virtual Reality viewers.

Refugees from Syria now constitute more than a sixth of the population of Lebanon, a small country that does not have the resources to cope with such a massive humanitarian crisis on its own (UNHCR, 2020). Effective response to this humanitarian crisis requires productive collaboration among countries around the world. In best practice, international and national aid agencies, supported by a broad range of implementing partners, leverage local expertise and resources in the collaborative design, development, and implementation of humanitarian response (VanRooyen, 2013).

The Virtual Exchange in Global Health thus provided each student group, comprising students from the U.S. and Lebanon, with an authentic challenge in a refugee camp in the Beqaa Valley in Lebanon. Student groups were advised to select a challenge from one of the following subject areas: primary/secondary education, mental health, reproductive health, or geriatric health. Student groups also collaborated with local partner institutions embedded in the community with working ties to relevant national and international organizations. Students in each group consulted with experts in both countries and worked with one another to develop and propose a detailed solution to a problem based on their shared investigation.

### Data gathering and analysis

For this qualitative case study, researchers gathered data through a detailed post-exchange survey, conducted immediately following completion of the Virtual Exchange program. To avoid leading students—to encourage them to frame their own experience as much as possible—researchers used open ended questions, for example, “Tell us about the virtual exchange with students from overseas. What are your thoughts about it?” or “How did the experience of viewing recordings in Virtual Reality differ from viewing traditional video?” (The complete student survey is available in the Appendix.) To interpret the resulting data, researchers began with a Grounded Theory methodology (Charmaz, 2006; Strauss & Corbin, 1998). Two researchers, one from the U.S. and one from Lebanon, coded survey responses to identify emergent themes. After three rounds of separately coding subsets of the data and comparing results, researchers achieved 93% agreement and from there separately coded the remaining responses.

Researchers documented the presence or absence of themes, as well as any repetition of themes, in each student’s response. By noting presence or absence, they could determine how broadly the theme was represented among the group of students. By noting repetition, they could see how deeply the thought or feeling was held. Among the emergent themes, they looked for correspondences to the bidirectionality of the programming, its focus on locally identified needs and priorities, and its structure to build local capacity to ensure sustainability. For example, almost half the students expressed a wish for more time to complete the assignment, but few expanded on that thought—breadth but not depth—with little connection to course themes. Conversely, a majority of students expressed appreciation for the opportunity to learn from counterparts with different backgrounds and highlighted this theme in responses to several different questions—both breadth and depth—with clear correspondence to the bidirectionality of the programming.

## Results

In their responses, students overwhelmingly expressed appreciation for the exchange. By a factor of more than ten to one, students wrote about what they valued in the experience, such as the opportunity to work collaboratively with overseas counterparts and to witness conditions in a refugee camp through Virtual Reality, in comparison to what they noted as difficulties, such as issues with inconsistent technology or scheduling across different time zones. Descriptions of difficulties were framed in a constructive manner, as ways to improve upon the experience.

### Bidirectionality of programming

Overall, students described the Virtual Exchange as “enriching”, “innovative”, and “unique”, especially for the opportunity that it provided to interact with overseas counterparts. Sixteen of the total 21 respondents explicitly remarked that a course with Virtual Exchange had distinct advantages over a traditional course without technology. The 16 students repeated this theme in response to different questions. Raters counted 32 separate occurrences in the total body of data, indicating both depth as well as breadth. One student from Lebanon wrote: “I realized that the project is very serious and not just theoretical … and would actually help the refugees in real life.”

In particular, 14 students commented favorably on the opportunity to learn from counterparts and advisors from different backgrounds; they repeated this idea for a total of 39 occurrences in the data, again indicating breadth and depth in the thought. In the words of one student from Lebanon:

LB: I think it’s a great opportunity for mutual gain. On one hand, one country gets acquainted with ... cultural norms and traditions which are often overlooked in the literature or lesser known. On the other hand, there’s an exposure to various approaches to a problem [that opens] dialogue.

The comment was echoed by this student from the U.S.:

US: The act of collaborating with my international team and having people on the ground to directly investigate the problems we were asking was the most valuable thing I gained from the course.

In these responses, students showed not only an appreciation of the bidirectionality of the Virtual Exchange program, but more specifically, a recognition of the complementary strengths of their overseas counterparts. Faculty routinely make this point in traditional lectures and accompanying slide presentations. In Virtual Exchange, by contrast, students experienced firsthand the benefits that diversity of perspective offers in a complex problem-solving process.

### Locally identified needs and priorities

Not everyone commented on Virtual Reality, but for those who did, the experience was notable. Eleven students made 50 comments on the impact of Virtual Reality to their learning, including 9 who made 13 specific comments that Virtual Reality gave them a sense of presence in the camps. Some students were especially moved by the experience. In the words of this student from the U.S.:

US: [Virtual Reality] definitely added to the experience and my understanding of the refugee camps because I could imagine myself walking in their shoes and viewing the size and layout of the tents ... You walk in the shoes of another and therefore you are more likely to empathize.

Or this student from Lebanon:

LB: It made the view more realistic as if I was with them, thus I felt more with what they are suffering from and increased my persistence to help them.

Here, Virtual Exchange and Virtual Reality led students not only to a cognitive understanding of locally identified of needs and priorities, but also to a greater sense of empathy towards refugees in the camps as well as a greater sense of urgency in responding to the crisis.

### Building local capacity to ensure sustainability

In a couple of cases, students in Lebanon explicitly stated that the program would help advance their career path, including this student from Lebanon:

LB: I realized how beneficial this experience was and how much it would help me advance in my career path.

More frequently, students framed this gain in terms of the relationships they built with their overseas counterparts. Twelve students made 24 comments on this theme, including this student from Lebanon:

LB: I was so excited to work with students abroad. I've noticed that such interaction will be fruitful and will help me later in my career as my network will increase with NGOs.

And this student from the U.S.:

US: Without the virtual exchange, we would not have had the humbling opportunity to get to know some of the most driven and accomplished MUBS students ... Their personal insights and opinions allowed me to better understand what life is like for them in Lebanon.

Thus, students framed building of local capacity and long-term sustainability in terms of the relationships they built with overseas counterparts.

The students’ work product also demonstrated the program’s contribution towards building local capacity to ensure sustainability. At the end of the program, students presented their analysis of problems in the camps, along with proposals for solutions, to a panel of experts from both the U.S. and Lebanon. All the students did outstanding work, but the judges singled out the work of one group in particular, whose project was suitable for implementation. The team identified mothers as a key demographic in refugee camps. Mothers tend to take primary responsibility for care of children and the elderly, who often suffer from depression in this context, and provide support to their husbands, whose ineligibility for work can also lead to depression. The students designed their program to build capacity, preparing mothers to navigate the rigors of living in a refugee camp, and to build community, connecting mothers in supportive networks to provide mutual assistance. With guidance from both universities, local implementation of this project began in September of 2019. Like most everything else, program activities were delayed by the pandemic, but students remain engaged and the program continues. The contribution of Virtual Exchange to sustainable global health interventions that build local capacity is clear.

Students also identified areas for improvement. Course facilitators put great effort to ensuring that there would be no technical difficulties, and for the most part, there were not. Yet audio problems crept in for a few of the sessions and had an unfortunate tendency to interfere with the flow of discussion and dialogue. Students generally wished for more project time, especially considering the difference in the time zones. One student in each country felt that the problems outweighed the benefits of the experience and would not recommend it to fellow students as implemented. In this student’s words, “the amount of effort that seemed to be desired was not in line with how many credits the course was” and the course “did not feel like a generative learning experience for me.” In all other responses (14) students said that they would recommend the course to fellow students, and tended to express their feelings in adamant terms:

LB: It was an amazing experience and it helped me to understand how people think about different issues and how they have different perspectives.

US: I would highly recommend to any other students taking a class where you can engage with people of different cultures, specifically through a collaboration project where you have to learn about another's culture in order to succeed and progress.

LB: I’m really happy that I participated in this course, learned a lot and made new friends. I will definitely participate again in any new projects or programs.

US: It was an excellent experience and such a fun opportunity – one of the most meaningful things I have done at Stanford, if not the most.

## Discussion

We developed this program aiming to show that Virtual Exchange in Global Health could provide a meaningful cross-cultural learning experience at substantially reduced costs and carbon footprint, demonstrating the potential of technology to expand access to socially responsible programming in the field of global health. The data that we collected provide confirming evidence that it can. The program was balanced and bidirectional, focused on locally identified needs and priorities, and structured to build capacity for students in both countries to ensure long-term sustainability.

In humanitarian response to the Syrian refugee crisis, and in response to other similar crises, different actors bring different kinds of expertise to bear. International actors typically bring technical expertise accumulated through repeated humanitarian relief efforts around the world. Local actors bring expertise on specific needs and particular context, which vary widely from place to place and is crucial to understand. In each case, different but overlapping expertise, resulting from diverse experience and perspective, are required for successful intervention. Through intelligent application of technology, students in the Virtual Exchange in Global Health were able to experience this interdependence. In addition to points of commonality that students discovered through the exchange—that they were roughly the same age, that they held a similar professional commitments and personal aspirations—they also came to recognize that they needed one another to be successful in their assignment, an experiential lesson in mutual value that spoke more authoritatively than lectures, texts, or recorded videos—and one that will serve them well as professionals.

In this case, rather than use technology to recreate what is possible in a traditional classroom, researchers used technology to transcend traditional teaching and provide students with a meaningful, cross-cultural learning experience at dramatically reduced cost and carbon footprint. This lesson has ramifications during the pandemic, when teachers are forced to use technology to conduct their classes, as well as after the pandemic, when faculty and students return to physical classrooms. The need for accessible cross-cultural learning in global health will remain; this study demonstrates the potential of Virtual Exchange to provide meaningful, cost-effective, collaborative learning for students across the globe.

## Conclusion

The program merits refinement and replication. In a next-stage pilot, researchers, faculty, and facilitators will respond to student feedback to enhance the experience. Researcher will also enrich data collection through diary studies and recording of key videoconference activities. Longer-term efforts will focus on scaling the program to benefit students of global health in the U.S. and around the world.

## Data Availability

The interview data generated and analyzed during the current study are not publicly available due to privacy concerns.

## Appendix: Open-ended survey questions

1. Tell us about the virtual exchange with students from overseas. What are your thoughts about it?
2. You could have taken this course without the online connection to students and faculty in another country. You could have learned about people in another country just from readings and lectures. How might that have been different?
3. Can you give us some examples to illustrate your thoughts about this?
4. When you first heard about interacting and working with students from another class in another country, what were your thoughts?
5. Did your feelings about the experience change or evolve as you got more into it, and if so how?
6. Tell us about working with students from the other class. What were your thoughts about that?
7. Tell us about the joint assignment. What were your thoughts about that?
8. What were the group interactions like on the joint assignment? Can you walk us through an example of how these interactions went?
9. Did you feel you had a chance to be heard? Can you give us some examples one way or the other?
10. Did you note any differences in perspective in your interactions with students from the other country? Can you give us some examples?
11. Tell us about the videoconferencing. What were the pros and cons of videoconferencing as a tool for the exchange?
12. Tell us about the Learning Management System (LMS). What were the pros and cons of LMS as a tool for the exchange?
13. Tell us about the recorded video lectures. What were the pros and cons of the recorded lecture as part of the experience?
14. Tell us about the joint class readings? What were the pros and cons of joint reading as part of the experience?
15. Tell us about viewing 360-degree video during this project. Were you able to view the recordings in a Virtual Reality (VR) viewer? What was your experience?
16. How did the experience of viewing recordings in Virtual Reality differ from viewing traditional video?
17. Has the 360-degree video technology added or not added to your appreciation of the experience of refugees in the camps, and if so, please explain?
18. Could Virtual Reality be used successfully to communicate conditions in refugee camps and other public health issues to others in your community or around the world? Why or why not?
19. Please tell us about any best experiences, worst experiences, or experiences that stood out the most.
20. Was there anything about the exchange experience that surprised you, and if so what?
21. Considering everything, what would you want to see changed if you did this again?
22. Considering everything, would you recommend this experience to another student, and why or why not?
23. Is there anything else we should know about your experience with virtual exchange?
